# High Tumour Cannabinoid CB_1_ Receptor Immunoreactivity Negatively Impacts Disease-Specific Survival in Stage II Microsatellite Stable Colorectal Cancer

**DOI:** 10.1371/journal.pone.0023003

**Published:** 2011-08-25

**Authors:** Sofia B. Gustafsson, Richard Palmqvist, Maria L. Henriksson, Anna M. Dahlin, Sofia Edin, Stig O. P. Jacobsson, Åke Öberg, Christopher J. Fowler

**Affiliations:** 1 Department of Pharmacology and Clinical Neuroscience, Pharmacology, Umeå University, Umeå, Sweden; 2 Department of Medical Biosciences, Pathology, Umeå University, Umeå, Sweden; 3 Department of Surgical and Perioperative Sciences, Surgery, Umeå University, Umeå, Sweden; Bauer Research Foundation, United States of America

## Abstract

**Background:**

There is good evidence in the literature that the cannabinoid system is disturbed in colorectal cancer. In the present study, we have investigated whether CB_1_ receptor immunoreactive intensity (CB_1_IR intensity) is associated with disease severity and outcome.

**Methodology/Principal Findings:**

CB_1_IR was assessed in formalin-fixed, paraffin-embedded specimens collected with a consecutive intent during primary tumour surgical resection from a series of cases diagnosed with colorectal cancer. Tumour centre (n = 483) and invasive front (n = 486) CB_1_IR was scored from 0 (absent) to 3 (intense staining) and the data was analysed as a median split i.e. CB_1_IR <2 and ≥2. In microsatellite stable, but not microsatellite instable tumours (as adjudged on the basis of immunohistochemical determination of four mismatch repair proteins), there was a significant positive association of the tumour grade with the CB_1_IR intensity. The difference between the microsatellite stable and instable tumours for this association of CB_1_IR was related to the CpG island methylation status of the cases. Cox proportional hazards regression analyses indicated a significant contribution of CB_1_IR to disease-specific survival in the microsatellite stable tumours when adjusting for tumour stage. For the cases with stage II microsatellite stable tumours, there was a significant effect of both tumour centre and front CB_1_IR upon disease specific survival. The 5 year probabilities of event-free survival were: 85±5 and 66±8%; tumour interior, 86±4% and 63±8% for the CB_1_IR<2 and CB_1_IR≥2 groups, respectively.

**Conclusions/Significance:**

The level of CB_1_ receptor expression in colorectal cancer is associated with the tumour grade in a manner dependent upon the degree of CpG hypermethylation. A high CB_1_IR is indicative of a poorer prognosis in stage II microsatellite stable tumour patients.

## Introduction

The G-protein coupled cannabinoid_1_ (CB_1_) receptors are most well known for their role in mediating the psychotropic effects sought after by recreational users of cannabis. However, CB_1_ receptors and their endogenous ligands anandamide (arachidonoylethanolamide) and 2-arachidonoylglycerol mediate a multitude of effects in the body including the regulation of pain, bone formation, energy homeostasis and gastrointestinal function [1]–[4]. In the human colon, CB_1_ receptors are found in the crypt epithelium as well as in subepithelial inflammatory cells, in smooth muscle of blood vessels and in submucosal plexus [5], [6], where they modulate, among other functions, the rate of intestinal transit and colonic propulsion [4].

In addition to the functions described above, the endocannabinoid system acts as a “damage limiting” system to mitigate the effects of pathological situations. This appears to be particularly true for the gastrointestinal endocannabinoid system. Thus, inflammation induced by agents such as 2,4-dinitrobenzene sulfonic acid, trinitrobenzene sulfonic acid, mustard oil or dextran sulfate sodium is more pronounced in CB_1_^−/−^ mice than in their wild type littermates, whilst treatment with a CB receptor agonist, or with compounds blocking the cellular removal and metabolism of endocannabinoids, alleviates the inflammation [7]–[11]. A CB_1_ receptor polymorphism (1359 G/A) is associated with a reduced susceptibility to ulcerative colitis in man [12], although to our knowledge it is not yet known how this single nucleotide polymorphism affects cannabinoid signalling. Aberrant crypt foci in the colon, an early pathological change in the adenoma-carcinoma sequence in colorectal cancer development, are formed as a result of azoxymethane treatment in mice, and the treatment is associated with an increase in the levels of 2-arachidonoylglycerol. Blockade of the metabolism of this endocannabinoid reduced the incidence of aberrant crypt foci, as did treatment with a CB receptor agonist [13] and, perhaps surprisingly, by treatment with a CB_1_ receptor inverse agonist [14].

Cannabinoids and endocannabinoids produce potentially useful effects upon cancer cell proliferation, motility and invasive behaviour (reviews, see [15], [16]). In colorectal cancer cell lines, both CB_1_-dependent and -independent effects of endogenous and/or synthetic cannabinoids upon cell viability have been reported [17]–[20]. In a genetic model of colorectal cancer progression (Apc^Min/+^ mice), animals lacking the CB_1_ receptor showed a greater number of small intestinal and colonic polyps than the corresponding CB_1_^+/+^ mice [21]. Colorectal cancer patients who are either homo- or heterozygous for the 1359 G/A CB_1_ receptor polymorphism show a shorter survival time than the G/G wild-type patients [22]. Finally, reduced expression of CB_1_ receptor mRNA and protein have been reported in colorectal cancer [17], [21], due at least in part to an increased rate of methylation of the promotor region of the receptor [21].

The above data are consistent with the suggestion that the endogenous cannabinoid system may be dysfunctional in colorectal cancer, and that such a dysfunction may affect disease severity and/or outcome. One way of investigating this possibility is to determine the level of CB_1_ receptor expression in a large cohort of well-characterised cases of colorectal cancer with long follow-up times. This has been undertaken in the present study. Given that colorectal cancer is highly heterogeneous, a particular focus has been made upon the relation of the CB_1_ immunoreactive intensities with key pathological/molecular components of the disease [23]: stage, tumour grade, microsatellite instability screening status, incidence of buds at the tumour front, and CpG island methylator phenotype (CIMP).

## Methods

### Ethics Statement

The research ethical committee at Umeå university hospital (Regional Ethical Review Board in Umeå, Sweden) approved the handling of tissue samples and patient data in the present study, including the procedure whereby patients verbally gave their informed consent. This consent was documented in each patient record, and this was considered by the ethical committee to be sufficient. In the database used for the analyses here, the tissue samples were given a case number and year, and the patient names were not indicated in the database.

### Patients

The formalin-fixed, paraffin-embedded samples used in the present study were obtained from tissue collected during primary tumour surgical resection of colorectal cancer (CRUMS (Colorectal cancer in Umeå study)). The samples were collected with a consecutive intent at the Department of Surgery, Umeå University Hospital, Sweden, during the period 1995–2003 and where possible the patients were followed for up to 113 months [24]. In addition to the clinico-pathological data reported in [24], data on the microsatellite stability/instability screening status (immunochemical determination of the expression of four mismatch repair proteins) and CIMP have been collected and previously reported [25]. Incidence of buds at the tumour front were evaluated as in [26]. Exclusion criteria were insufficient or unavailable tumor tissue sample and insufficient clinical information. All in all, 487 cases were scored for either tumour centre or tumour front CB_1_ receptor immunoreactivity (see below). The clinical information in the database for these patients was as follows: median age 71 years (range 26–96, n = 487, of which 269 were males and 218 females); cancer location in right colon 31.1%, left colon 31.1%, rectum 37.8% (n = 482); disease stage I 15.5%, II 39.3%, III 21.0%, IV 24.2% (n = 476); tumour grade well/well-moderately differentiated 48.75%, moderate-poor/poorly differentiated 51.25% (n = 480); microsatellite stable 85.0%, microsatellite instable 15.0%; (n = 473); CIMP status negative 50.4%, low 37.2%, high 12.4% (n = 484). Further, 82.4% (of 483 cases) did not receive preoperative radiotherapy (either 5×5 Gy or 25×2 Gy); 75.0% (of 476 cases) had radical surgery; and 13.7% (of 475 cases) received adjuvant chemotherapy.

### Measurement of CB_1_ receptor immunoreactivity (CB_1_IR) in the tumour tissue

The paraffin-embedded tissue sections were deparaffinized and rehydrated before antigen retrieval in a pressure cooker (2100 retriever, Biocare Medical) in Diva Decloaker (Biocare Medical). Samples were subsequently placed in a Ventana semiautomated immunostaining machine (Ventana Medical Systems Inc., Tucson, AZ). The CB_1_ receptor antibody (AbCam cat. no. 23703, AbCam plc, Cambridge, UK, diluted 1∶50) and the secondary components (iVIEW DAB Detection Kit, Ventana Medical Systems Inc.) were then added. The antibody, a rabbit polyclonal raised to an peptide corresponding to C terminal amino acids 461–472 of the human CB_1_ receptor and which cross-reacts with the human, mouse and rat CB_1_ receptor according to data from the manufacturers, has been shown previously by researchers in Umeå (including the corresponding author) to produce the appropriate pattern of staining in human cerebellum, but not to produce immunoreactivity in forebrains from CB_1_^−/−^ mice [27]. An ExPasy Blast (http://expasy.org/tools/blast/) of the peptide sequence gave CB_1_ and its two splice variants as the only hits in man. In several other species, CB_1_ receptors were again identified, the only non-CB_1_ hits being from proteins termed “Uncharacterized protein [Gene: CNR1]” (from pig, dog and chicken) as well as “Putative uncharacterized protein [Gene: PANDA_015085] - *Ailuropoda melanoleuca* (Giant panda)” and “Chromosome 14 SCAF15003, whole genome shotgun sequence fragment” in *Tetraodon nigroviridis* (Green pufferfish).

CB_1_IR was assessed by one investigator (SBG) who was blinded to the clinical status of the patients. The samples were graded on the basis of the dominant CB_1_ receptor immunoreactive intensity (CB_1_IR) in the tumour interiors and in the tumour invasive fronts and scored from 0 (absent) to 3 (intense) for the cells. When all samples had been scored, the investigator repeated the procedure (without access to the previous scores) and then compared the scores on the two runs. Cases where the scores were divergent were then assessed a third time, again without access to the previous scores. For the tumour front samples scored, for example, there were 53 occasions where the first and second scores disagreed, due mainly to erroneous scores from the initial stage of the first run. The final scores were then entered by another investigator (CJF) into the database for analysis.

### Statistical evaluations

Kaplan-Meier survival analyses, Fisher's exact test and χ^2^ tests were undertaken using the statistical package built into the GraphPad Prism 5 computer programme for the Macintosh (GraphPad Software Inc., San Diego, CA, USA). Cox proportional-hazards and binary logistic regression analyses were conducted using IBM SPSS statistics 19 software for the Macintosh (IBM Inc., Somers, NY, USA). For survival analyses, disease-specific events were defined as death with known disseminated or recurrent disease (“†_ca_”). Death from other causes was censored, as were the cases where the patient was still alive at the date of last follow-up. The duration of event-free survival is defined as the time from diagnosis until either the date of colorectal cancer death, death of other causes, or if no death occurred, until the date of last follow-up.

## Results

### CB_1_IR immunohistochemistry

**Figure 1:**
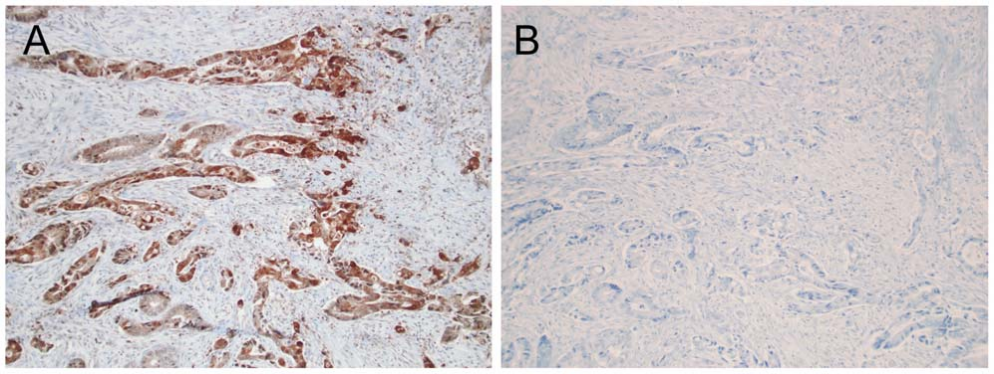
CB_1_ receptor immunoreactivity in tumour samples. Sections from the same case were used in the presence (Panel A) and absence (Panel B) of the primary antibody. Objective magnification is 10×.

Initial studies were undertaken using the same batch of the antibody as in a previous study by researchers in Umeå using tissue microarrays from prostate cancer samples [27]. We found that at a dilution of 1∶300, CB_1_IR was found in the epithelial cells of the crypts, with scattered positivity in subepithelial inflammatory cells (Fig. S1A), a finding consistent with studies of CB_1_IR in the normal colon [5], [6]. An example of tumour tissue stained with this batch is shown in Fig. 1B. Due to limited amounts of antibody remaining, we used a later batch of the AbCam antibody (batch 761993) for the main study, and found that a lower dilution (1∶50) was required for good immunostaining. An example of staining is seen in Fig. 1A, with a corresponding serial section without primary antibody shown in Fig. 1B.

The best specificity test for immunochemistry is considered to be the use of knockout controls [28]. We found that appropriate immunochemical staining was seen in the brains of wild-type mice, whereas this was absent from brains of CB_1_^−/−^mice (Fig. S2). Ashton [29] has recently argued that optical density histograms are useful in distinguishing between a change in the pattern of immunolabelling (as should be seen for a true loss of signalling in a knockout) compared to a reduction in labelling intensity (which would raise a question as to the validity of the antibody). Optical density histograms of our wild-type and CB_1_^−/−^ tissues are clearly different (best seen for the higher magnification slides, Fig. S2), consistent with good antibody specificity [29].

A common way of testing for antibody specificity is to investigate preadsorbtion of the antibody with the immunising peptide, although the usefulness of this measure has been questioned [30]. Nevertheless, a series of experiments using preincubation of the antibody with the immunising peptide (AbCam, Cat. No. 50542) were also undertaken. However, preincubation with the immunising peptide greatly increased the observed immunoreactivity rather than blocked it, and gave a rather random pattern of immunoreactive staining (data not shown). The immunising peptide (amino acid sequence MSVSTDTSAEAL) has two negatively charged amino acids (aspartate and glutamate), five polar amino acids (three serine, two threonine) and no positively charged amino acids. It is well known that negatively charged peptides bind to glass, and we conclude that the washing procedure in the Ventana technique, while being sufficient to provide good specificity *per se* (as seen in the knockout mice), is not sufficient to remove antibody-bound positive control peptide adhering non-specifically to glass and/or to zwitterionic lipids.

### Distribution of CB_1_IR scores in the tumour centre and fronts

A total of 483 (tumour interior) and 486 (tumour front) cases were scored for CB_1_IR intensity and entered into the database. Both plasma membrane and cytoplasmic CB_1_IR was scored, so the values represent the total pool of CB_1_ receptors. Nuclear staining of CB_1_IR (found in 42 cases) was not scored. The frequency distributions of the CB_1_IR for the tumour centre and fronts were similar, with a score of 0 being returned for 77 and 60 cases; a score of 1 for 196 and 185 cases; a score of 2 for 140 and 156 cases; and a score of 3 for 70 and 85 cases (numbers are tumour centre and fronts, respectively). There was no significant difference in the distribution pattern of the two sets of scores (p>0.1, χ^2^ test). It was noted that the scores for the two regions were not always the same for a given case. Indeed, for the 482 cases scored for both tumour centre and front, the scores were the same in only 319 (66%) of the cases, being higher in the tumour front in 110 (23%) cases and higher in the tumour centre in 53 (11%) cases. In consequence, throughout this study, both tumour centre and tumour front scores have been analysed separately. Non-malignant tissue was not scored, but in general its level of immunoreactivity appeared to be lower rather than higher than seen for the tumour tissue.

### Association of CB_1_IR with patient characteristics at surgery

**Figure 2:**
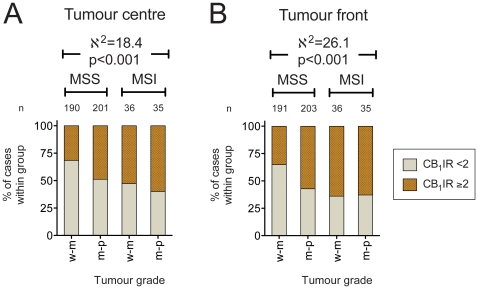
Influence of the tumour histological grade and MSI screening status upon CB_1_IR in colorectal cancer. Panel A, tumour centre; Panel B, tumour front. The data are grouped according to tumour histological grade (w-m, well/well-moderately differentiated; m-p, moderate-poor/poorly differentiated) and microsatellite stability (MSS, stable; MSI, instable). The χ^2^ and hence p values are for the data grouped as a 4×2 matrix (where the 2 is the CB_1_IR). n refers to the total number of cases for each bar.

In view of the frequency distribution of the CB_1_IR, the analyses were conducted using a simple median split, i.e. CB_1_IR<2 and ≥2. Using the entire data set to search for variables, a binary logistic regression with parameters gender, site (i.e. right colon, left colon, rectum), radiotherapy (prior to surgery), disease stage, tumour histological grade (i.e. differentiation), tumour type (i.e. mucinous or non-mucinous), microsatellite instability screening status [stable (MSS) or instable (MSI)], amount of lymphocytes at the tumour front and the frequency of tumour buds (small aggregates of tumour cells at the tumour invasion front, [31]) was conducted. Of these variables, only the tumour histological grade (p<0.005) and microsatellite instability screening status (p<0.05) were significantly associated with the CB_1_IR (data not shown). These effects can simply be visualised by dividing the dataset into four groups on the basis of the two significant parameters (Fig. 2). The majority of the cases were classified as MSS, and the detailed CB_1_IR distributions for the MSS cases who did not receive radiotherapy prior to surgery are summarised in Table 1. In both the tumour centre and tumour fronts, there are more cases with a CB_1_IR≥2 for MSS cases with moderate-poor/poor tumour differentiation than with well/well-moderately differentiated tumours. In the MSI cases, this effect of the tumour histological grade is not seen, and the cases have a similar CB_1_IR distribution to the moderate-poor/poorly differentiated MSS cases (Fig. 2). Further analysis of the 62 cases with MSI scored for CB_1_IR who did not receive radiotherapy prior to biopsy revealed no significant association of the tumour centre or front CB_1_IR with either disease stage (here stages I and II were combined since only 4 cases with stage I were scored for tumour centre CB_1_IR), tumour grade, whether the tumours were mucinous or non-mucinous, incidence of lymphocytes at the tumour front or CIMP status (p>0.1, Fisher's exact test or χ^2^ test, as appropriate, data not shown). For gender, the p values for the CB_1_IR distributions were 0.069 and 0.18 for tumour centre and front, respectively (Fisher's exact test), and for the incidence of buds at the tumour front, the p values (χ^2^ test) were 0.086 and 0.073, respectively (data not shown).

**Table 1:** CB_1_IR in tumour interiors for microsatellite stable (MSS) cancers: correlation with patient characteristics for patients not receiving radiotherapy prior to surgery.

	Tumour centre				Tumour front			
Parameter	n	CB_1_IR<2	CB_1_IR≥2	p	n	CB_1_IR<2	CB_1_IR≥2	p
Age (y)^a^	318	72 [26–96]	74 [35–89]	0.14^a^	321	72 [26–93]	72 [35–96]	0.49^a^
*Gender*								
Males	175	107 (61%)	68 (39%)	0.91^b^	178	104 (58%)	74 (42%)	0.43^b^
Females	143	86 (60%)	57 (40%)		143	77 (54%)	66 (46%)	
*Site*								
Right colon	92	57 (62%)	35 (38%)	0.40^c^	95	48 (51%)	47 (49%)	0.22^c^
Left colon	136	77 (57%)	59 (43%)		135	74 (55%)	61 (45%)	
Rectum	87	57 (66%)	30 (34%)		87	55 (63%)	32 (37%)	
*Disease stage*								
I	40	27 (68%)^d^	13 (33%)^d^	0.31^c^	40	23 (58%)^d^	17 (43%)^d^	0.57^c^
II	123	74 (60%)	49 (40%)		125	74 (59%)	51 (41%)	
III	59	30 (51%)	29 (49%)		60	29 (48%)	31 (52%)	
IV	89	57 (64%)	32 (36%)		89	49 (55%)	40 (45%)	
*Tumour grade*								
w-m	170	116 (68%)	54 (32%)	0.0052^b^	171	113 (66%)	58 (34%)	0.0001^b^
m-p	143	75 (52%)	68 (48%)		145	64 (44%)	81 (56%)	
*Tumour type*								
Mucinous	41	26 (63%)	15 (37%)	0.86^b^	42	20 (48%)	22 (52%)	0.24^b^
Non- mucinous	273	165 (60%)	108 (40%)		275	159 (58%)	116 (42%)	
*Lymphocytes (at TF)*								
Low no.	168	99 (59%)	69 (41%)	0.64^b^	171	88 (51%)	83 (49%)	0.069^b^
High no.	145	90 (62%)	55 (38%)		145	90 (62%)	55 (38%)	
*Buds (at TF)*								
None	20	11 (55%)	9 (45%)	0.66^c^	20	8 (40%)	12 (60%)	0.054^c^
1–9	132	83 (63%)	49 (37%)		133	85 (64%)	48 (36%)	
10–19	79	45 (57%)	34 (43%)		79	38 (48%)	41 (52%)	
≥20	75	49 (65%)	26 (35%)		78	46 (59%)	32 (41%)	
*CIMP status*								
Negative	173	106 (61%)	67 (39%)	0.83^c^	174	99 (57%)	75 (43%)	0.85^c^
Low	128	78 (61%)	50 (39%)		129	74 (57%)	55 (43%)	
High	15	8 (53%)	7 (47%)		16	8 (50%)	8 (50%)	

Abbreviation: TF, tumour front; CIMP, CpG island methylator phenotype. w-m, well/well-moderately differentiated, m-p, , moderate-poor/poorly differentiated.

a Data for age is given as medians with range, and the p value was from a Mann-Whitney U-test.

b p values determined by Fisher's exact test.

c p values determined by χ^2^ test.

d The rounding up of the % (e.g. 67.5% → 68%) gives the sum total of 101% for the tumour centre and front data.

**Figure 3:**
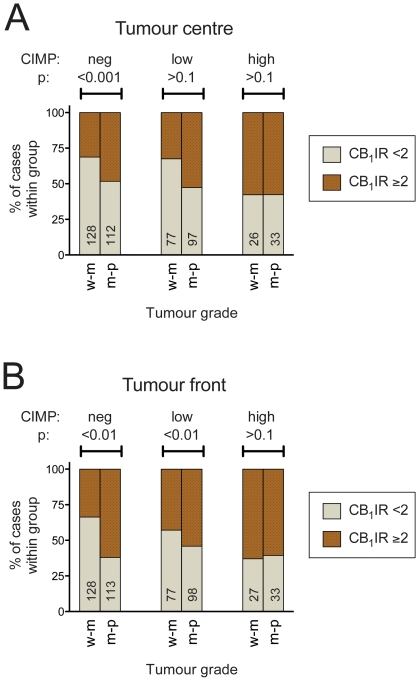
Patients with CIMP-high tumours have CB_1_IR levels that are not dependent upon the tumour grade. Panel A, tumour centre; Panel B, tumour front. The data are grouped according to tumour histological grade (w-m, well/well-moderately differentiated; m-p, moderate-poor/poorly differentiated) and the CIMP status. Of the 473 cases scored for tumour centre CB_1_IR, 389 were classified as MSS, 71 as MSI and 13 not classified in the data base. The corresponding numbers for the tumour front CB_1_IR were 392, 71 and 13, respectively. The p values are for Fisher's exact test. The total (i.e. CB_1_IR<2 and ≥2) number of cases is shown enclosed within each bar.

One major difference between MSS and MSI is the greater incidence of a high degree of CpG island methylation in the latter [32]. In our data set, of the 467 cases scored for microsatellite instability screening status, CIMP status and tumour centre CB_1_IR, the CIMP distributions were: MSS (n = 396), negative 225 (57%), low 153 (39%) and high 18 (4.5%); MSI (n = 71), negative 15 (21%), low 17 (24%) and high 39 (55%) (p<0.0001, χ^2^ test). When the CIMP status of the samples was added into the binary logistic regression of the whole dataset with the parameters described above, the significant effect of the tumour histological grade was retained (p<0.005) for both tumour centre and front CB_1_IR, but the effect of the microsatellite instability screening status was lost for the tumour centres (p>0.1) but not for the tumour fronts (p<0.05) (data not shown). In order to investigate this further, the data for the samples was divided into the three CIMP groups: negative, low and high regardless of microsatellite instability screening status or whether the patients had received radiotherapy prior to surgery. There was a clear influence of CIMP status on the results, where the effect of the tumour histological grade was seen in the cases with a CIMP-negative score but not in the cases with a CIMP-high score, the CIMP-low cases being somewhere in the middle (Fig. 3). This analysis did not take into account the microsatellite instability screening status of the cases in order to achieve sufficient group sizes. However, a similar pattern is seen when the MSS and MSI cases are analysed separately, although as a caveat it should be pointed out that some of the group sizes are very small (Fig. S3).

### Association of CB_1_IR with disease outcome

The cases in the database had been followed for up to 113 months [24] allowing the influence of the CB_1_IR score at diagnosis upon disease outcome to be determined. Using the entire dataset (i.e. even those cases where CB_1_IR was not scored, a univariate Cox proportional hazards regression analysis on the entire dataset indicated that the administration of radiotherapy prior to surgery was, unsurprisingly, associated with disease outcome (Exp(B) 0.60 [95% CI 0.40–0.91] p<0.05) where the number of cases not receiving/receiving radiotherapy was 441/102. The Cox proportional hazards regression analysis is a test used to determine the contribution of the parameter(s) under investigation upon disease-specific survival without making assumptions as to the nature of the survival curve. The measure Exp(B), sometimes called the hazards ratio, indicates the change in risk as the parameter under investigation is changed from the default parameter (here no radiotherapy) to the test parameter (here radiotherapy). In this case, the radiotherapy reduced the risk of death due to the disease since the Exp(B) value was significantly lower than unity. In order to remove the influence of this parameter, the CB_1_IR data was analysed only for the cases that did not receive radiotherapy. In addition, the MSS and MSI cases were analysed separately.

**Figure 4:**
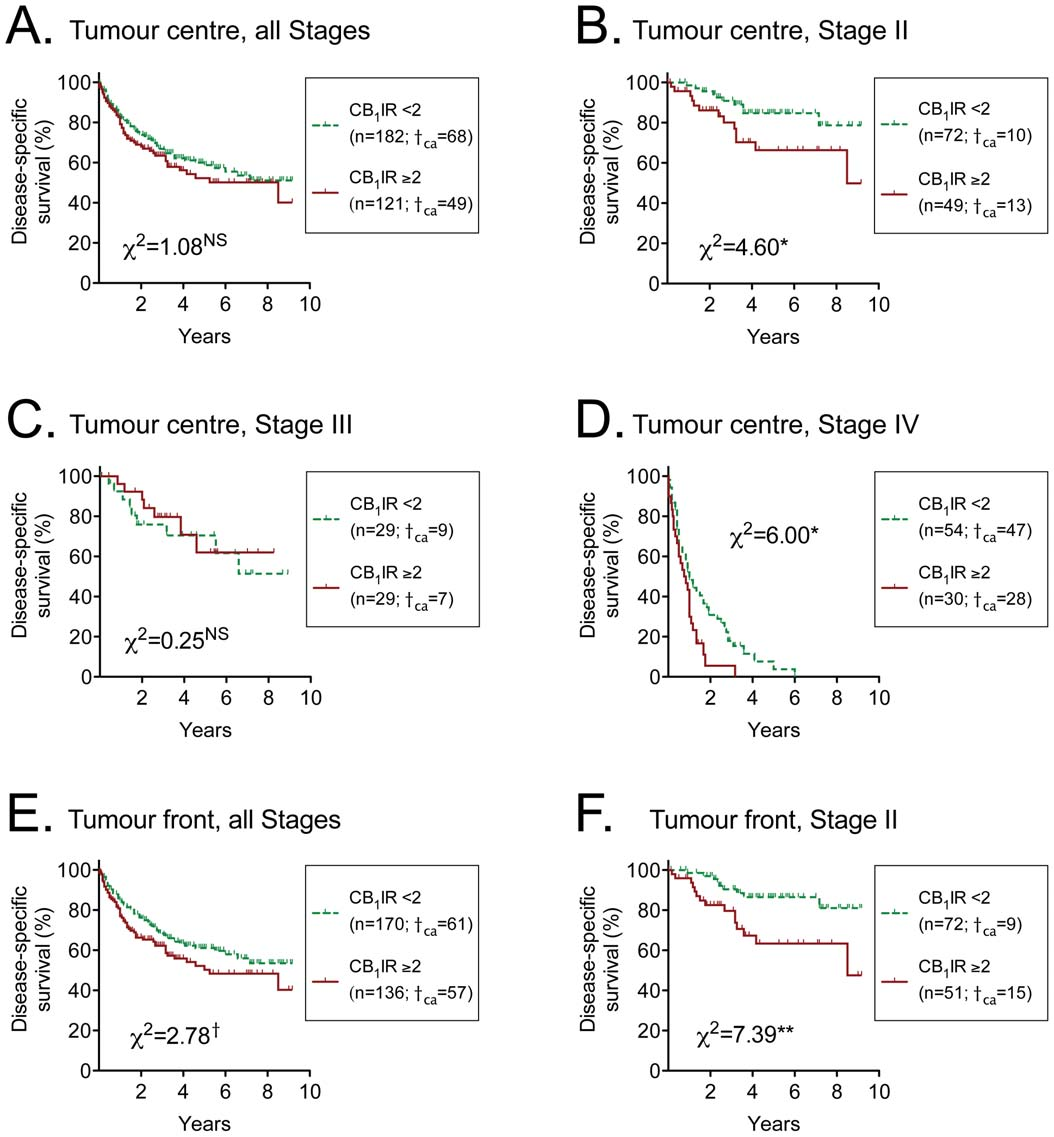
Influence of CB_1_IR scores at surgery upon disease-specific survival. Kaplan-Meier plots of the disease-specific survival for the tumour regions and disease stages shown. Shown in the figures are the number of cases (n) followed by the number who died as a result of the colorectal cancer (†_ca_). The χ^2^ value given in the figures is from the log-rank (Mantel- Cox) test comparing the two curves; **p<0.01, *p<0.05, ^†^0.05<p<0.1; ^NS^p>0.1. The corresponding χ^2^ values for tumour front stages III and IV were 0.24^NS^ and 1.44^NS^, respectively.

For the MSS cases, a univariate Cox proportional hazards regression analysis failed to show a significant effect of either tumour interior or front CB_1_IR upon disease-specific survival. However, when a bivariate analysis was undertaken with disease stage as the second parameter, a highly significant contribution of CB_1_IR was seen (Table 2). This was confirmed in Kaplan-Meier survival plots: for the entire data set, there was no significant contribution of the tumour interior CB_1_IR (Fig. 4A), whereas in tumour stage II (where the tumours have infiltrated the muscularis propria of the colon or rectum, but have not given rise to lymph node metastasis), and in tumour stage IV (where the tumours have spread to other organs), cases with a CB_1_IR≥2 have a poorer disease-specific survival than cases with a CB_1_IR<2 (Figs. 4B and D; definitions of disease staging given in [33]). No difference was seen for tumour stage III, although it should be borne in mind that this is a highly heterogenous group, that the number of cases was smaller than for disease stages II and IV and that subgroup analyses suffer from low power. There were only three patients diagnosed with stage I colorectal cancer (where the tumour has only infiltrated the submucosal layer of the colon or rectum and has not spread to the lymph nodes or other organs) who died as a result of their cancer during the follow-up period, precluding analysis of the influence of CB_1_IR upon disease-specific outcome in this patient group. In the case of the tumour front CB_1_IR, the significant effect upon tumour outcome in the stage II patients was also seen, but not in the stage IV patients (Fig. 4 and data not shown, respectively). In the stage II cases, the 5 year probabilities of event-free survival were: 85±5 and 66±8%; tumour interior, 86±4% and 63±8% for the CB_1_IR<2 and CB_1_IR≥2 groups, respectively. It can be noted that some of the stage II cases died within one month of surgery. However, when these cases were excluded, the significant contribution of CB_1_IR to the disease-specific outcome was retained (data not shown). Upon further subdivision of the stage II cases according to cancer site, a significant contribution of both tumour centre and front CB_1_IR upon disease-specific survival was seen for the rectal cancers, but not for the colon cancers, although the direction (i.e. poorer survival for a high CB_1_IR) was the same. It is important to note, however, that interpretation of results with these subgroups are limited by a low power and are not supported by the Cox analyses (see below).

**Table 2:** Cox proportional-hazards regression analyses for microsatellite stable (MSS) cancers; influence of disease stage, tumour grade and number of tumour front buds.

		Tumour centre		Tumour front	
Variable	Cat. value	n	Exp(B) [95%CL]	n	Exp(B) [95%CL]
*Univariate analyses*					
CB_1_IR	<2 (1)	182	1	170	1
	≥2 (2)	121	1.21 [0.84–1.75]^NS^	136	1.36 [0.94–1.95]†
*Bivariate analyses*					
CB_1_IR	<2 (1)	182	1	170	1
	≥2 (2)	121	1.72 [1.18–2.53]**	136	1.45 [1.01–2.09]*
Disease Stage	I (1)	40	1	40	1
	II (2)	121	2.50 [0.75–8.35]^NS^	123	2.67 [0.80–8.86]^NS^
	III (3)	58	4.08 [1.19–14.0]*	59	4.14 [1.20–14.2]*
	IV (4)	84	41.0 [12.7–132]***	84	38.0 [11.8–122]***
*Multivariate analyses*					
CB_1_IR	<2 (1)	180	1	166	1
	≥2 (2)	118	1.60 [1.08–2.37]*	135	1.32 [0.91–1.92]^NS^
Disease Stage	I (1)	37	1	37	1
	II (2)	121	2.29 [0.69–7.63]^NS^	123	2.38 [0.72–7.92]^NS^
	III (3)	57	3.68 [1.07–12.7]*	58	3.66 [1.06–12.6]*
	IV (4)	83	35.3 [10.9–114]***	83	32.4 [10.0–104]***
Tumour grade	w-m (1)	162	1	163	1
	m-p (2)	136	1.44 [0.98–2.13]†	138	1.55 [1.05–2.27]*
*Multivariate analyses*					
CB_1_IR	<2 (1)	175	1	162	1
	≥2 (2)	111	1.85 [1.22–2.82]**	128	1.67 [1.12–2.49]*
Disease Stage	I (1)	37	1	37	1
	II (2)	116	2.61 [0.78–8.71]^NS^	118	2.62 [0.79–8.71]^NS^
	III (3)	55	3.79 [1.10–13.1]*	56	3.82 [1.11–13.2]*
	IV (4)	78	41.1 [12.6–134]***	79	38.2 [11.8–124]***
Tumour grade	w-m (1)	157	1	158	1
	m-p (2)	129	1.36 [0.92–2.02]^NS^	132	1.39 [0.94–2.05]†
Buds (at TF)	None (0)	20		20	
	1–9 (1)	123	1.49 [0.58–3.82]^NS^	124	1.97 [0.76–5.08]^NS^
	10–19 (2)	73	1.25 [0.48–3.27]^NS^	73	1.52 [0.59–3.96]^NS^
	≥20 (3)	70	2.78 [1.08–7.16]*	73	3.54 [1.35–9.31]*

Abbreviations: TF, tumour front; w-m, well/well-moderately differentiated; m-p, , moderate-poor/poorly differentiated; Cat. value, categorical value. Exp(B) refers to the increase in the odds as a result of an increase in the “unit” (shown in brackets in the categorical value column). Significance levels:

***p<0.001,

**p<0.01,

*p<0.05,

† 0.5>p>0.1,

NS p>0.1.

In a separate univariate analysis, the Exp(B) value (with 95% confidence limits) for the differentiation state parameter was 2.08 [1.44–3.00], p<0.001. For a bivariate analysis with disease stage and differentiation state, the Exp (B) value (with 95% confidence limits) for the differentiation state parameter was 1.60 [1.10–2.33], p<0.05. The level of significance was retained when no. of TF buds was added as a third parameter. Finally, in multivariate analyses with disease stage, no. of TF buds and CB_1_IR score, the Exp(B) value (with 95% confidence limits) for tumour centre and tumour front CB_1_IR scores were 1.97 [1.31–2.96], p<0.01 and 1.81 [1.23–2.68], p<0.01, respectively.

The fact that in the MSS patients the CB_1_IR is associated with the tumour histological grade at surgery may of course mean that the association described above is simply a reflection of the influence of the tumour histological grade upon disease outcome. This possibility was investigated using multivariate Cox proportional hazards regression analyses (Table 2), where it was found that the tumour histological grade provided additive prognostic information to that seen with the tumour centre CB_1_IR when the disease stage was also included as a parameter. In contrast, the tumour front CB_1_IR parameter lost significance. However, when the incidence of buds at the tumour front was also included (in itself a prognostic factor), the influence of the tumour histological grade upon disease outcome was reduced, whereas the influence of both tumour center and front CB_1_IR was significant (Table 2). These data would suggest that the prognostic significance of the tumour centre and possibly also tumour front CB_1_IR is not secondary to its association with the tumour histological grade. Further analysis indicated that the prognostic significance of CB_1_IR remained when the CIMP status, gender and tumour region were included in the multivariate analysis (tumour centre, Exp(B) 1.77 [95% CL 1.16–2.72, p<0.01; tumour front Exp(B) 1.67 [95% CL 1.11–2.50] p<0.05).

For the MSI cases, no conclusions could be drawn as to whether CB_1_IR impacted upon disease-specific survival in stage II cases, simply because of the 32 cases that fell into this category (after exclusion of cases receiving radiotherapy prior to surgery), only one died of the cancer during the follow-up period.

## Discussion

The present study was motivated by data from both cultured cells and patient samples suggesting that a dysfunctional endocannabinoid signalling system is involved either in the pathogenesis and/or as a consequence of colorectal cancer [13], [14], [17], [21], [22]. At the outset it is worth commenting upon the fact that both cell surface and cytoplasmic CB_1_ receptors were scored. CB_1_ receptors are often regarded as cell surface receptors, but it is well established in many cells and tissues that they have been found intracellularly [34]–[37], as would be expected for receptors that internalise following sustained agonist stimulation [38], [39]. It has been suggested that these intracellular receptors are active and couple to extracellular signal-regulated kinase [35], although other authors have not seen intracellular co-localisation of CB_1_ receptors with Gα subunits [39]. Naturally-occurring ligands for CB_1_ receptors are highly lipophilic, and thus the plasma membrane is not a barrier to their cellular penetration. Extracellular signal-regulated kinase is an important signalling molecule, and has been implicated in antiproliferative effects of cannabinoids in a number of different cancer cell lines [40], so it is not unreasonable (and technically considerably less difficult) to score the combined intensity from plasma membrane and intracellular CB_1_ receptors. There are three main results in from the study, and these are discussed in turn.

### 1. Tumour centres and invasive fronts have different CB_1_IR intensities in one third of cases investigated

For both tumour centres and invasive fronts, a gamut of CB_1_IR scores from absent (0) to pronounced (3) were seen. Although there was no significant difference between the CB_1_IR distribution patterns for the two regions, one third of the cases had a score for the tumour invasive front that was different from that for the corresponding tumour centre CB_1_IR. One possible explanation for this difference is that the tumour front is an area of intense immunological and inflammatory activity [41], and it can be hypothesised that constituents of the tumour front microenvironment affect the transcription of CB_1_ receptors in this region. One potential candidate in this respect is the cytokine interleukin-4 (IL-4), given that it can increase CB_1_ receptor expression [42], and that both IL-4 and IL-4 receptors are found in colon tumours [43], [44]. It would be clearly of interest to determine whether other constituents of the tumour microenvironment affect CB_1_ receptor transcription.

### 2. The tumour histological grade is associated with CB_1_IR in a manner modulated by the CIMP status

For MSS cases, well/well-moderately differentiated tumours distribute with the ratio of CB_1_IR<2∶≥2 among the cases of approximately 2∶1, whilst the ratio is close to 1∶1 for the cases with moderate-poor/poorly differentiated tumours at surgery. In the MSI cases, no such difference is seen and the cases have a ratio near 1∶1. MSI cases are characterised by a high incidence of mutated DNA microsatellite markers as a result of a loss of DNA mismatch repair, and differ from MSS cases not only in terms of tumour characteristics and gene expression profiles [45] but also in the survival rates and responses to chemotherapy [46]. It is thus perhaps not surprising that the MSS and MSI cases have different CB_1_IR distributions. However, a major difference between MSS and MSI cases are the relative incidences of CIMP-negative, low and high [32], and our analysis suggests that the CIMP status rather than the microsatellite stability is a prime determinant of the association of tumour grade with CB_1_IR. It is notable that for all cases with a negative CIMP status and moderate-poorly differentiated tumours, the relative proportion of cases with CB_1_IR≥2 is similar to that seen for well/well-moderately and for moderate-poor/poorly differentiated CIMP-high tumours. This would suggest that the shift in CB_1_IR distribution seen with histological grade is brought about along the same pathway as the shift seen with CIMP-high, so that the effects are not additive.

With respect to the effects of DNA hypermethylation upon CB_1_ receptor expression, Wang et al. [21] investigated in a series of 13 cases the methylation status of 39 cytosine and guanine-rich DNA segments (“CpG islands”) in the region (−212 to +140) of the start site for transcription of the *Cnr1* gene (the gene responsible for the CB_1_ receptor). These authors found hypermethylation of these sites in the colorectal tumours, ranging from cases with a single action at position +108 to cases with hypermethylation in ≥15 sites. They reported that the hypermethylation resulted in *Cnr1* gene silencing [21]. Although their study shows an effect in the opposite direction to the apparent effect of a CIMP-high score seen here, it is important to stress that the determination of CIMP in our study was based on a validated eight gene screening panel [25] and is thus an indication that the tumours have a high frequency of hypermethylated genes in general, whereas Wang et al [21] focused on methylation of sites directly relevant to the *Cnr1* gene. It is possible that one or several genes that are commonly inactivated by hypermethylation in CIMP-high tumours have downstream effects upon the transcription and/or turnover of the CB_1_ receptor, and this produces the results seen here, or alternatively that the pattern of *Cnr1* hypermethylation in colorectal cancer is rather different from that picked up in the CIMP screen. In the latter situation, a case with a low level of CB_1_ promotor methylation but a high CIMP score (or *vice versa*) would be classified differently in the two studies, so it is not surprising that the results are divergent. In future studies, it would clearly be of interest to investigate *Cnr1* hypermethylation in our tissue material, to be able to distinguish between these alternatives.

### 3. A high CB_1_IR is associated with a poorer disease-specific survival in patients with stage II MSS colorectal cancer

From the introduction, it might have been expected that a high CB_1_ tumour receptor expression would be beneficial to the patients, whereas the opposite was found to be the case, at least for the patients with stage II MSS tumours at surgery. To our knowledge, only three studies have been undertaken to investigate the prognostic value of CB_1_IR in solid tumours. In hepatocellular carcinoma, the 35 cases with an undetectable or faint CB_1_IR showed a significantly poorer disease-free survival than the 29 cases with a moderate or intense CB_1_IR [47]. Interestingly, the distribution of CB_1_IR was also associated with histological grade, with 20/34 of the cases with well/well-moderately differentiated tumours showing a high CB_1_IR while only 9/30 cases with moderate-poor/poorly differentiated tumours showed a high CB_1_IR [47]. The other two studies, one in pancreas cancer and one in prostate cancer, are consistent with the present study. In the pancreatic cancer study, two cohorts were used. In the first cohort (n = 37), a composite scale of immunoreactive intensity×distribution was used, and cases with a high score were found to have a significantly shorter median survival than those with a low score [48]. The same result was seen in a second cohort (n = 53) measuring CB_1_ receptor mRNA expression with quantitative RT-PCR. In the prostate cancer study (conducted in Umeå using the same antibody as in the present study, albeit a different batch), a composite score was again used, and the disease-specific survival was significantly poorer for the 192 cases with a CB_1_IR equal to or above the median (15 year probability of event-free survival 50±5%) than for the 77 cases with a CB_1_IR below the median (15 year probability of event-free survival 78±7%) [26].

It is of course naïve to assume that the influence of the CB_1_IR score is going to be the same regardless of the cancer in question, but the present study would suggest that hepatocellular carcinoma, rather than colorectal cancer, is the “odd cancer out”. The question nevertheless remains as to why a high, rather than a low, CB_1_ expression should be associated with a poorer disease-specific survival. One possible explanation has been furnished by a recent study using cultured astrocytoma cells transfected with CB_1_ receptors [49]. In that study, the authors selected clones with different CB_1_ receptor expression levels and found that at a low receptor expression, the receptors coupled primarily to extracellular signal-regulated kinases, and that activation of the CB_1_ receptors led to apoptosis. In contrast, at a high level of CB_1_ receptor expression, activation of the receptors led additionally to the activation of the Akt survival pathway, and cannabinoids only produced apoptosis when this pathway was inhibited [49]. It is of course a long way from studies in transfected cells to the situation in solid tumours, but the postulation that a high CB_1_ receptor expression results in the switch from a pro-apoptotic to a predominantly pro-survival pathway would mean that the local endocannabinoid tone no longer acts to limit the damaging influence of the tumour but rather to exacerbate it and thereby result in a poorer prognosis for the patient. The hepatocellular carcinoma data [47] can be incorporated into this admittedly speculative hypothesis by suggesting that the cancers defined as high CB_1_IR do not have a sufficiently high level of expression to trigger the switch in these cells.

A final note concerns the potential of CB_1_IR as a prognostic marker to aid treatment decisions in cancer. In prostate cancer, CB_1_IR looks to be a very promising marker that provides additive prognostic information to that supplied by other variables such as the Gleason score and the tumour stage [27], [50]. For colorectal cancer, the situation is less promising, since prognostic significance was not across the board, but unmasked in the MSS cases when the disease stage was also taken into consideration. Nonetheless, given that patients with stage II colorectal cancers are a patient group where treatment decisions are difficult and better prognostic markers are needed [51], the present data warrant further investigation into the potential usefulness of CB_1_IR as a prognostic marker to aid such treatment decisions in stage II MSS colorectal cancer.

## Supporting Information

Figure S1 **CB_1_ receptor immunoreactivity in non-malignant and adenocarcinoma samples.** Panel A, non-malignant tissue; Panel B adenocarcinoma tissue, both stained using the antibody batch used in [27]. Objective magnification is 10×.
(TIF)

Figure S2 **CB_1_ receptor immunoreactivity in forebrain samples from wild-type and CB_1_ receptor knockout mice.** Panels A and B show the immunoreactivity from wild-type and CB_1_ receptor knockout mice, respectively. Objective magnification is 1.25×. The tiff image from the selected areas was imported into Adobe Photoshop (version CS4 for the Macintosh) and the colour histograms were captured. Panels C (wild-type) and D (CB_1_ receptor knockout) show immunoreactivity from different forebrain tissue slides to those in Panels A and B, at a higher objective magnification (20×). The colour histograms are for the whole images. The paraffin embedded, formalin-fixed mouse tissue was kindly provided by Drs. Beat Lutz and Giacomo Mancini, Department of Physiological Chemistry, Johannes Gutenberg-University Mainz, Germany.
(TIF)

Figure S3 **Division of CB_1_IR scores according to tumour grade, CIMP status and microsatellite stability screening status.** Panel A, tumour centre; Panel B, tumour front. The data are grouped according to tumour grade (w-m, well/well-moderately differentiated; m-p, , moderate-poor/poorly differentiated) and microsatellite stability (MSS, stable; MSI, instable) and the CIMP status. P values were determined using Fisher's exact test. The total (i.e. CB_1_IR<2 and ≥2) number of cases is shown enclosed within each bar.
(TIF)
