# Supplementary figures and images for: High Tumour Cannabinoid CB1 Receptor Immunoreactivity Negatively Impacts Disease-Specific Survival in Stage II Microsatellite Stable Colorectal Cancer
Source: PLoS One. 2011 Aug 25;6(8):e23003. doi: 10.1371/journal.pone.0023003 (PMC3161987; doi:10.1371/journal.pone.0023003)

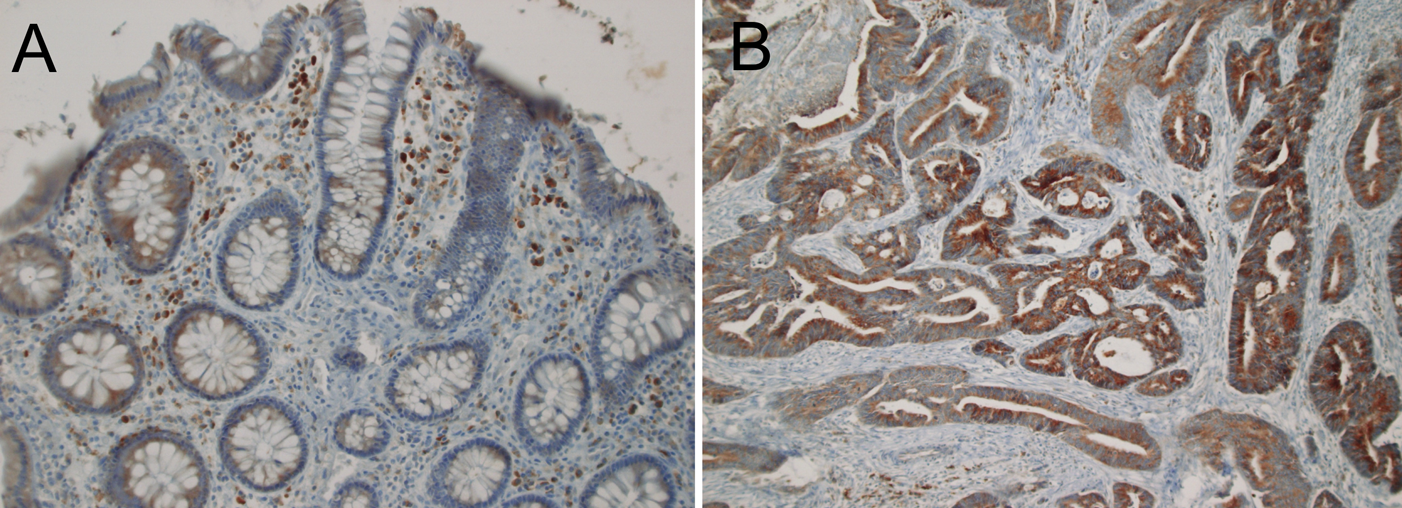

Supplement: Figure S1 — CB1 receptor immunoreactivity in non-malignant and adenocarcinoma samples. Panel A, non-malignant tissue; Panel B adenocarcinoma tissue, both stained using the antibody batch used in [27]. Objective magnification is 10×. (TIF) [file pone.0023003.s001.tif]

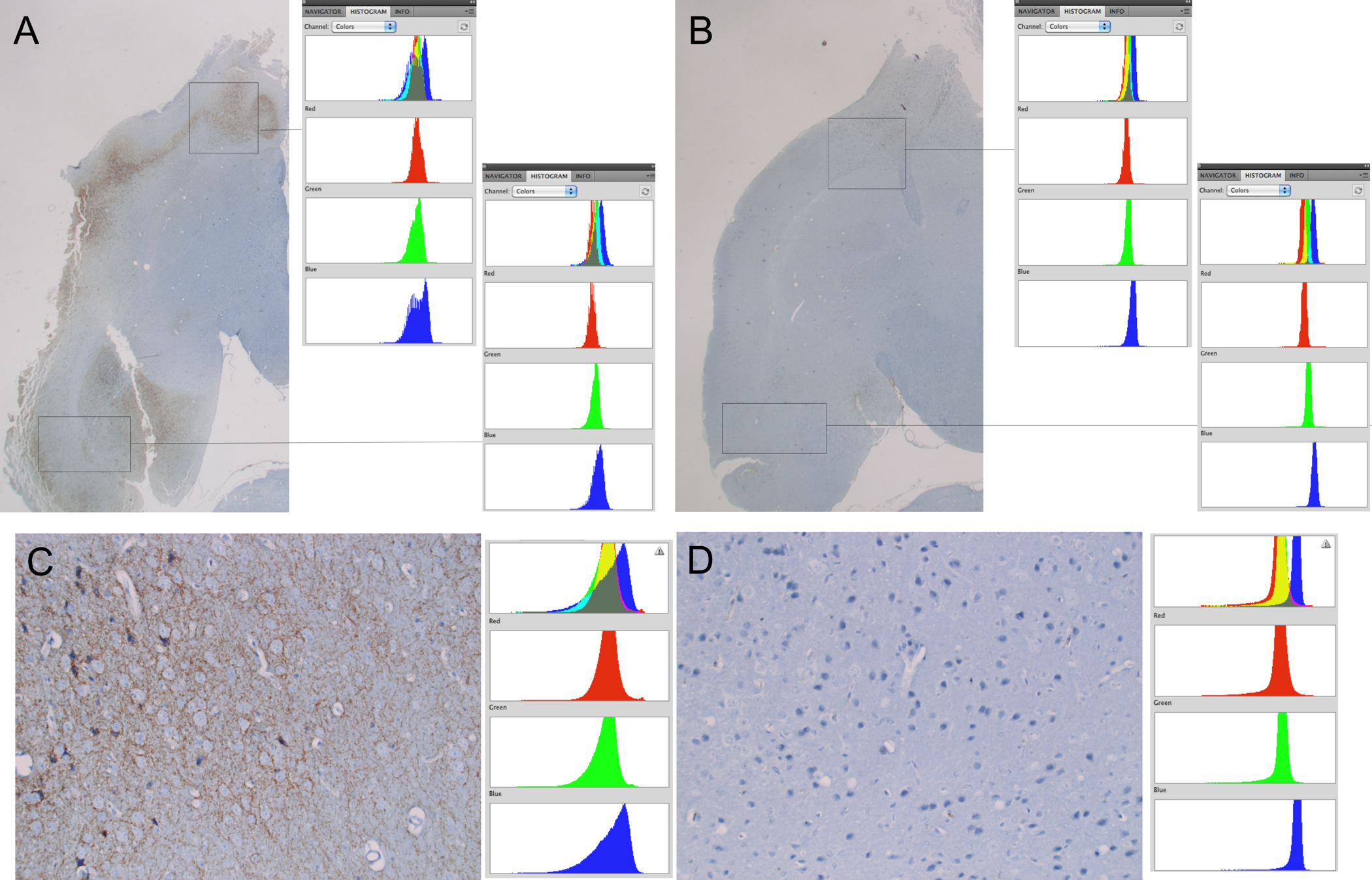

Supplement: Figure S2 — CB1 receptor immunoreactivity in forebrain samples from wild-type and CB1 receptor knockout mice. Panels A and B show the immunoreactivity from wild-type and CB1 receptor knockout mice, respectively. Objective magnification is 1.25×. The tiff image from the selected areas was imported into Adobe Photoshop (version CS4 for the Macintosh) and the colour histograms were captured. Panels C (wild-type) and D (CB1 receptor knockout) show immunoreactivity from different forebrain tissue slides to those in Panels A and B, at a higher objective magnification (20×). The colour histograms are for the whole images. The paraffin embedded, formalin-fixed mouse tissue was kindly provided by Drs. Beat Lutz and Giacomo Mancini, Department of Physiological Chemistry, Johannes Gutenberg-University Mainz, Germany. (TIF) [file pone.0023003.s002.tif]

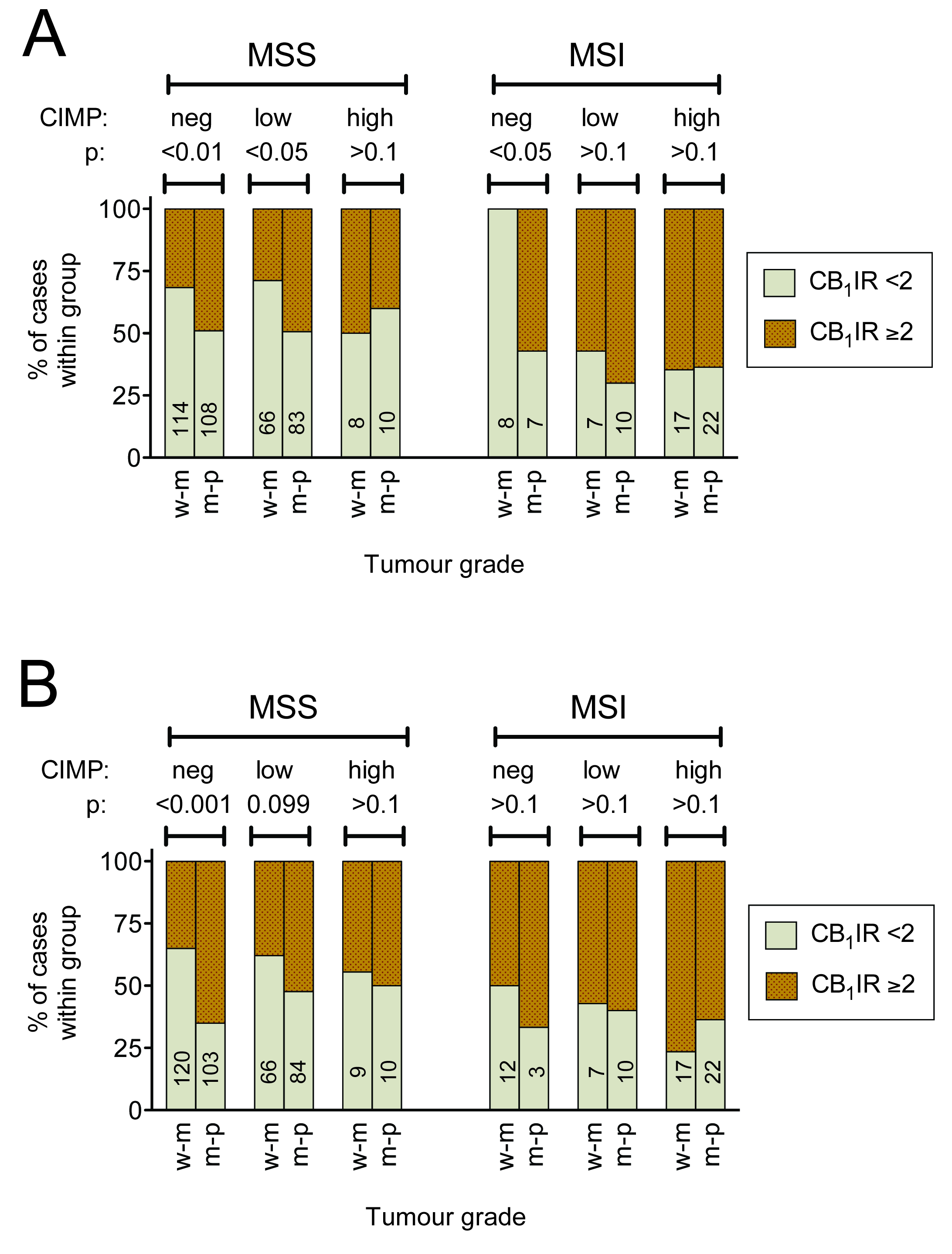

Supplement: Figure S3 — Division of CB1IR scores according to tumour grade, CIMP status and microsatellite stability screening status. Panel A, tumour centre; Panel B, tumour front. The data are grouped according to tumour grade (w-m, well/well-moderately differentiated; m-p, , moderate-poor/poorly differentiated) and microsatellite stability (MSS, stable; MSI, instable) and the CIMP status. P values were determined using Fisher's exact test. The total (i.e. CB1IR<2 and ≥2) number of cases is shown enclosed within each bar. (TIF) [file pone.0023003.s003.tif]
